# Tubular MYDGF Slows Progression of Chronic Kidney Disease by Maintaining Mitochondrial Homeostasis

**DOI:** 10.1002/advs.202409756

**Published:** 2024-11-26

**Authors:** Xiaohan Liu, Yang Zhang, Youzhao Wang, Yujie Yang, Zhe Qiao, Ping Zhan, Huiying Jin, Qianqian Xu, Wei Tang, Yu Sun, Yan Zhang, Fan Yi, Min Liu

**Affiliations:** ^1^ Department of Pharmacology School of Basic Medical Sciences Shandong University Jinan 250012 China; ^2^ Department of Pharmacy The Second Hospital Cheeloo College of Medicine Shandong University Jinan 250033 China; ^3^ Jincheng General Hospital Jincheng 048006 China; ^4^ Department of Organ Transplantation Qilu Hospital of Shandong University Jinan 250012 China; ^5^ National Key Laboratory for Innovation and Transformation of Luobing Theory Key Laboratory of Cardiovascular Remodeling and Function Research Chinese Ministry of Education and Chinese Ministry of Health Qilu Hospital Shandong University Jinan 250012 China

**Keywords:** chronic kidney disease, mitochondrial homeostasis, MYDGF, tubular epithelial cells

## Abstract

Mitochondrial dysfunction is a key event driving the maladaptive repair of tubular epithelial cells during the transition from acute kidney injury to chronic kidney disease (CKD). Therefore, identifying potential targets involved in mitochondrial dysfunction in tubular epithelial cells is clinically important. Myeloid‐derived growth factor (MYDGF), a novel secreted protein, plays important roles in multiple cardiovascular diseases, but the function of MYDGF in tubular epithelial cells remains unknown. In the present study, it is found that MYDGF expression is significantly reduced in the cortex of the kidney, especially in the proximal tubules, from mice with CKD. Notably, lower expression of MYDGF is observed in tubules from patients with CKD and the level of MYDGF correlated with key factors related to kidney fibrosis and estimated glomerular filtration rate (eGFR) in patients with CKD. Tubule‐specific deletion of *Mydgf* exacerbates kidney injury in mice with CKD; however, *Mydgf* overexpression attenuates kidney fibrosis by remodeling mitochondrial homeostasis in tubular epithelial cells. Mechanistically, renal tubular MYDGF positively regulates the expression of isocitrate dehydrogenase 2 (IDH2), restores mitochondrial homeostasis, and slows CKD progression. Thus, this study indicates that MYDGF derived from tubules may be an effective therapeutic strategy for patients with CKD.

## Graphical abstract

Myeloid‐derived growth factor (MYDGF) is decreased in proximal tubules from patients with chronic kidney disease (CKD) and correlates with key factors related to kidney fibrosis and estimated glomerular filtration rate. MYDGF deficiency in tubules exacerbates kidney injury in CKD mice. MYDGF overexpression upregulates isocitrate dehydrogenase 2 (IDH2), restores mitochondrial homeostasis, and slows CKD progression, suggesting that MYDGF is an attractive therapeutic target for CKD.

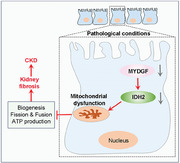

## 1 Introduction

Chronic kidney disease (CKD), defined as a persistent abnormality in kidney structure or function,^[^ ^1^ ^]^ is a public health threat owing to its high incidence and low awareness rate. Kidney fibrosis is the common pathway for the progression of different types of CKD to end‐stage renal disease (ESRD),^[^ ^2^ ^]^ which is a complex and highly dynamic process involving various renal parenchymal and interstitial cells.^[^ ^3^ ^]^ Among the components of the kidney, tubular epithelial cells are vital in regulating electrolyte transport, acid‐base homeostasis, and endocrine functions, and consume the vast majority of energy within the kidney.^[^ ^4^ ^]^ Consequently, tubular epithelial cells are sensitive to energy metabolism which is highly dependent on mitochondrial homeostasis.^[^ ^5^ ^]^ Mitochondrial abnormalities and dysfunction are key events in the maladaptive repair of tubular epithelial cells during the transition from acute kidney injury to CKD.^[^ ^6^ ^]^ Therefore, clarifying the underlying mechanisms and identifying potential targets related to mitochondrial dysfunction in tubular epithelial cells may be promising for developing effective therapeutic strategies for CKD.

Myeloid‐derived growth factor (MYDGF) is originally recognized as a secreted protein encoded by an open reading frame on chromosome 19 (C19orf10).^[^ ^7^ ^]^ Although MYDGF exhibits high sequence conservation across all vertebrate species,^[^ ^8^ ^]^ it does not belong to any known cytokine or growth factor family based on its primary amino acid sequence, indicating that it may have other biological functions. It is known that MYDGF derived from bone marrow cells, particularly monocytes and macrophages, protects against myocardial infarction,^[^ ^7^ ^]^ pressure overload‐induced heart failure,^[^ ^9^ ^]^ atherosclerosis,^[^ ^10^ ^]^ interstitial tissue damage,^[^ ^11^ ^]^ and diabetic kidney disease.^[^ ^12^ ^]^ However, emerging evidence has also revealed new aspects of MYDGF function in parenchymal cells. In injured neonatal hearts, MYDGF derived from endothelial cells, rather than macrophages, promoted cardiomyocyte proliferation and improved heart regeneration.^[^ ^13^ ^]^ In the kidney, the expression of MYDGF in renal tubules was higher than that in macrophages in healthy mice based on the data from the publicly available single‐cell datasets, suggesting that tubular MYDGF might play an important role in maintaining kidney homeostasis.^[^ ^14^ ^]^ However, the expression pattern and the role of tubular MYDGF in CKD remain unclear. In addition, the association of MYDGF with patients with CKD needs to be further investigated. In this study, we observed the level of MYDGF correlated with key factors related to kidney fibrosis and estimated glomerular filtration rate (eGFR) in patients with CKD. Using tubule‐specific deletion of *Mydgf* mice, we demonstrated that *Mydgf* deficiency promoted tubular injury by disrupting mitochondrial homeostasis in mice with various CKD models, suggesting that MYDGF is an attractive therapeutic target for CKD.

## 2 Results

### 2.1 MYDGF Expression was Significantly Reduced in Tubules of Mice with CKD

**Figure 1:**
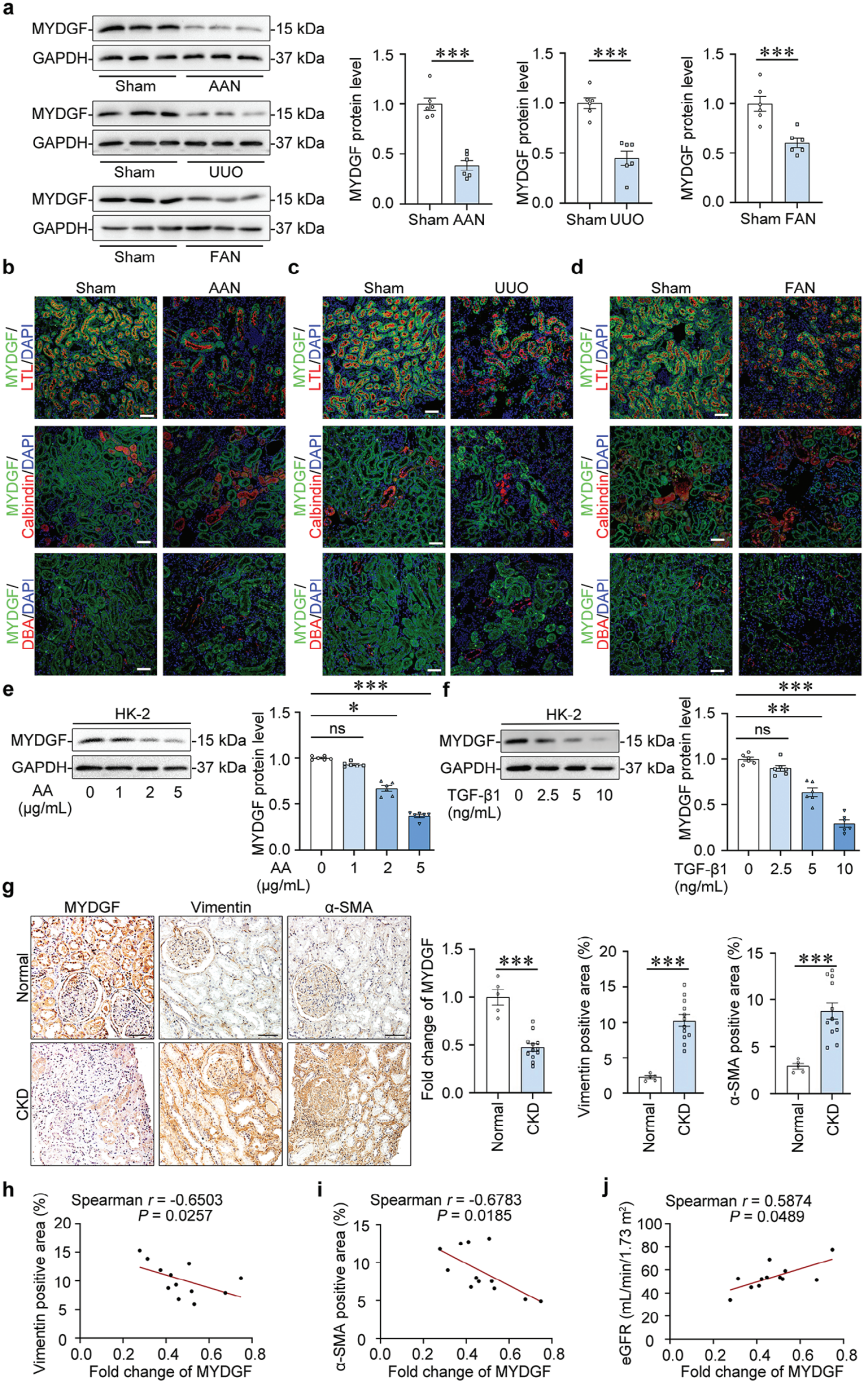
MYDGF was significantly reduced in tubules from mice and patients with CKD. a) Protein levels of MYDGF in the cortex of the kidney from AAN, UUO, and FAN mice. ^***^ *p* < 0.001. (n = 6 mice per group). b) MYDGF expression in tubules from AAN mice. Lotus tetragonolobus lectin (LTL) was used as a proximal tubular marker; calbindin D28k was used as a marker for distal convoluted tubule; dolichos biflorus agglutinin (DBA) was used as a marker for collecting duct. Scale bar, 50 µm. c) MYDGF expression in tubules from UUO mice. Scale bar, 50 µm. d) MYDGF expression in tubules from FAN mice. Scale bar, 50 µm. e) Protein levels of MYDGF in human tubule epithelial cells (HK‐2) with aristolochic acid (AA) treatment for 48 h. ^*^ *p* < 0.05, ^***^ *p* < 0.001. (n = 6 biologically independent experiments). f) Protein levels of MYDGF in human tubule epithelial cells (HK‐2) with transforming growth factor‐beta 1 (TGF‐β1) treatment for 48 h. ^**^ *p* < 0.01, ^***^ *p* < 0.001. (n = 6 biologically independent experiments). g) Photomicrographs and quantifications of MYDGF, Vimentin, and α‐SMA staining in kidney sections of patients with CKD. Scale bar, 50 µm. ***p < 0.001. (n = 5 for normal participants, n = 12 for patients with CKD). h) Correlation between MYDGF expression and the degree of Vimentin staining in all participants with CKD. (n = 12). i) Correlation between MYDGF expression and the degree of α‐SMA staining in all participants with CKD. (n = 12). j) Correlation between MYDGF expression and eGFR in all participants with CKD. (n = 12). Data are expressed as mean ± SEM (a,e–g). Two‐tailed Student's unpaired *t*‐test analysis (a,g). One‐way ANOVA followed by Tukey's post‐test (e,f). Spearman's correlation coefficient *r* with two‐tailed *P*‐value (h–j).

Using different animal models of CKD, we found that MYDGF expression was reduced in the cortex of the kidney of mice with aristolochic acid nephropathy (AAN), unilateral ureter obstruction (UUO), folic acid nephropathy (FAN) and bilateral renal ischemia‐reperfusion injury (BIRI) (**Figure** **1**a; Figure S1a, Supporting Information). Immunofluorescence staining showed that the level of MYDGF was significantly decreased in the proximal tubules (Figure 1b–d; Figure S1b–d, Supporting Information). In vitro, aristolochic acid (AA, Figure 1e), transforming growth factor‐beta 1 (TGF‐β1, Figure 1f), hypoxia or folic acid (FA, Figure S1e,f, Supporting Information) treatment reduced the expression of MYDGF in human tubule epithelial cells (HK‐2 cells), a proximal tubular cell line. Importantly, MYDGF reduction in the tubules was also confirmed in patients with CKD (Figure 1g), and the level of MYDGF correlated with Vimentin, α‐SMA, and eGFR (Figure 1h‐j), suggesting that tubular MYDGF is involved in kidney fibrosis and dysfunction in CKD.

### 2.2 Tubular MYDGF Protected Against Kidney Injury in Mice with AAN

**Figure 2:**
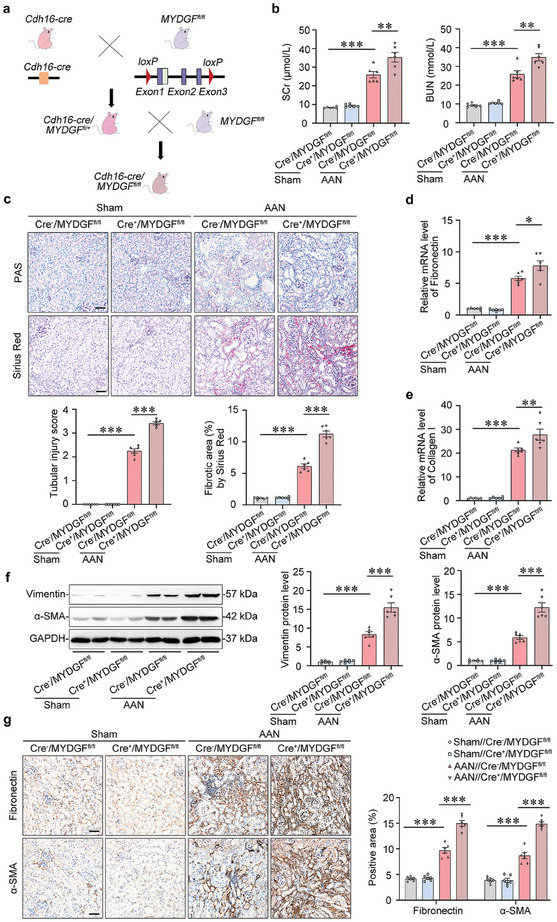
Tubule‐specific deletion of *Mydgf* exacerbated kidney injury in mice with AAN. a) Generation of conditional knockout mice in which *Mydgf* is specifically ablated in tubular cells by using *Cre–loxP* recombination system. b) Serum creatinine (SCr) and blood urea nitrogen (BUN) in different groups of mice. ^**^ *p* < 0.01, ^***^ *p* < 0.001. (n = 6 mice per group). c) PAS staining and Sirius Red staining were performed to assess kidney injury and fibrosis. Scale bar, 50 µm. ^***^ *p* < 0.001. (n = 6 mice per group). d) Relative mRNA level of *Fibronectin* in the cortex of the kidney from AAN mice. ^*^ *p* < 0.05, ^***^ *p* < 0.001. (n = 6 mice per group). e) Relative mRNA level of *Collagen I* in the cortex of kidney from AAN mice. ^**^ *p* < 0.01, ^***^ *p* < 0.001. (n = 6 mice per group). f) Representative Western blot gel documents and summarized data showed the relative protein levels of Vimentin and α‐SMA in the cortex of the kidney from different groups of mice. ^***^ *p* < 0.001. (n = 6 mice per group). g) Photomicrographs and quantifications of Fibronectin and α‐SMA staining in the kidneys from different groups of mice. Scale bar, 50 µm. ^***^p < 0.001. (n = 6 mice per group). Data are expressed as mean ± SEM (b–g). Two‐way ANOVA followed by Tukey's post‐test (b–g).

Based on the reduction in MYDGF expression in the tubules, tubule‐specific *Mydgf* knockout mice were generated by crossing *Mydgf^fl/fl^* mice with *Cdh16‐Cre* mice (**Figure** **2**a). The efficiency of *Mydgf* knockout in mice was confirmed by tail genotyping (Figure S2a, Supporting Information) and a significant decrease in MYDGF expression in the cortex of the kidney (Figure S2b, Supporting Information). All mice were viable and fertile. *Cre^+^/Mydgf^fl/fl^* mice did not show any physiological changes, including in body weight, kidney weight, heart rate, and blood pressure under normal conditions (Table S1, Supporting Information). However, tubule‐specific deletion of *Mydgf* increased the levels of serum creatinine (SCr) and blood urea nitrogen (BUN, Figure 2b), exacerbated tubular atrophy and extracellular matrix deposition in mice with AAN, based on periodic acid‐Schiff (PAS) and Sirius Red staining (Figure 2c). At the molecular level, *Mydgf* deficiency increased the expression of markers associated with fibrosis, including Fibronectin, Collagen I, Vimentin, and α‐SMA in the kidneys of mice with AAN (Figure 2d–g).

**Figure 3:**
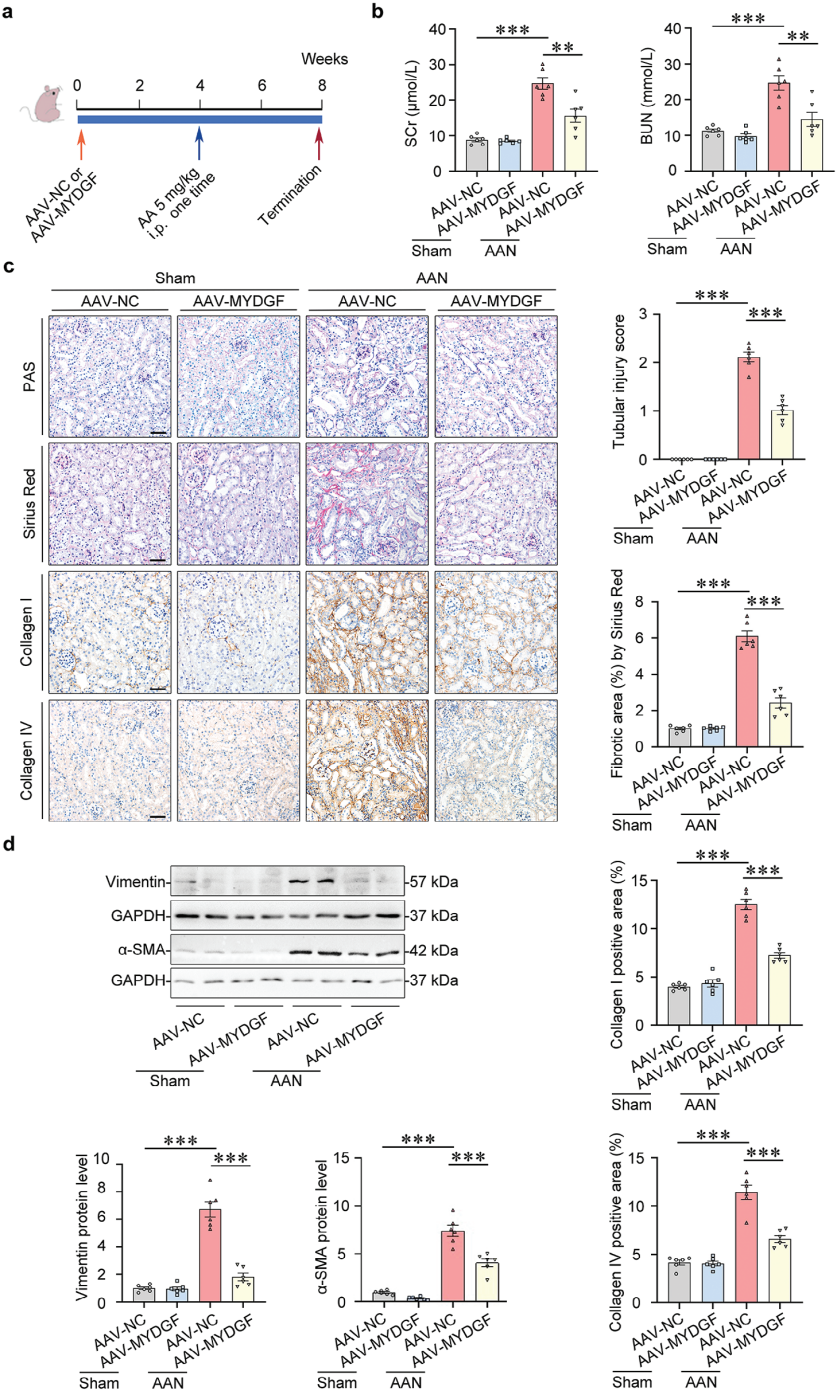
Overexpression of *Mydgf* attenuated kidney injury in mice with AAN. a) Schematic diagram shows the experimental procedure. b) Serum creatinine (SCr) and blood urea nitrogen (BUN) in different groups of mice. ^**^ *p* < 0.01, ^***^ *p* < 0.001. (n = 6 mice per group). c) PAS staining and Sirius Red staining were performed to assess kidney injury and fibrosis. Photomicrographs and quantifications of Collagen I and Collagen IV staining were performed to assess kidney fibrosis. Scale bar, 50 µm. ^***^ *p* < 0.001. (n = 6 mice per group). d) Representative Western blot gel documents and summarized data showed the relative protein levels of Vimentin and α‐SMA in the cortex of the kidney from different groups of mice. ^***^ *p* < 0.001. (n = 6 mice per group). Data are expressed as mean ± SEM (b–d). Two‐way ANOVA followed by Tukey's post‐test (b–d).

To examine the genetic therapeutic efficiency of targeting tubular *Mydgf* in mice with AAN, we delivered tubule‐specific AAV serotype 9 harboring *Mydgf* (pAAV9‐Ksp‐cadherin‐*Mydgf*) via a tail vein injection into mice (**Figure** **3**a). The upregulation of MYDGF in the kidneys of mice with AAV9 transfection (Figure S3a, Supporting Information) reduced the levels of serum creatinine and blood urea nitrogen (Figure 3b), attenuated tubular injury and tubulointerstitial fibrosis in mice with AAN, which was confirmed by the reduced Collagen I and Collagen IV, Vimentin and α‐SMA levels (Figure 3c,d). In vitro, overexpression of *MYDGF* (Figure S3b, Supporting Information) reduced the expression of Collagen I and α‐SMA in HK‐2 cells with AA treatment (Figure S3c, Supporting Information).

### 2.3 MYDGF Maintained Mitochondrial Homeostasis in Tubular Epithelial Cells

**Figure 4:**
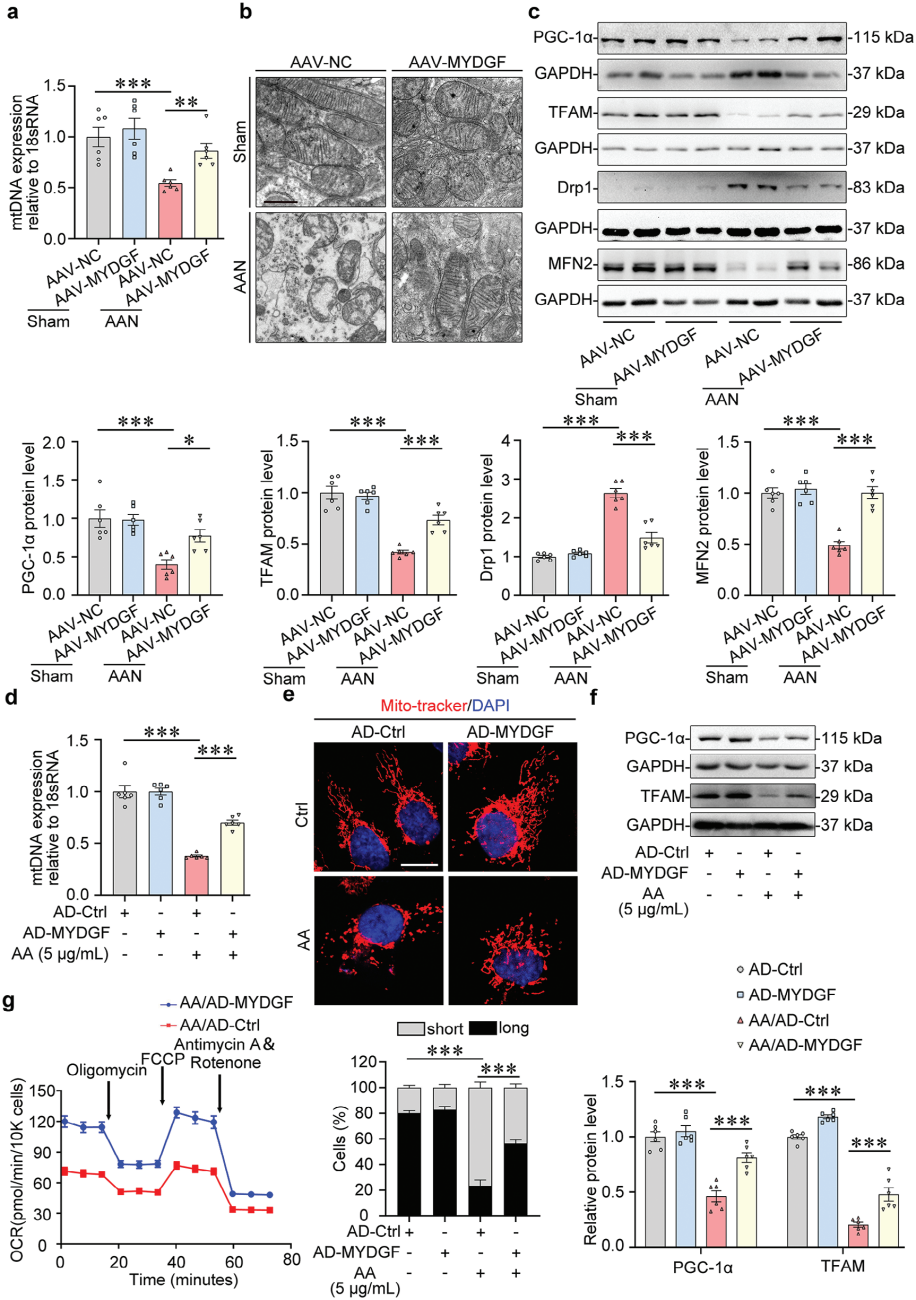
MYDGF maintained mitochondrial homeostasis in tubular epithelial cells. a) Quantitative analysis of mtDNA in the cortex of the kidney from different groups of mice. ^**^ *p* < 0.01, ^***^ *p* < 0.001. (n = 6 mice per group). b) Representative photomicrographs of mitochondria in different groups of mice by transmission electron microscopy (TEM) analyses. Scale bar, 1 µm. c) Representative Western blot gel documents and summarized data showed the relative protein levels of PGC‐1α, TFAM, Drp1, and MFN2 in the cortex of the kidney from different groups of mice. ^*^ *p* < 0.05, ^***^ *p* < 0.001. (n = 6 mice per group). d) Quantitative analysis of mtDNA in HK‐2 with aristolochic acid (AA) treatment. ^***^ *p* < 0.001. (n = 6 biologically independent experiments). e) Representative confocal microscopy images of mitochondria stained by Mito‐tracker in different groups of HK‐2 cells. Scale bar, 10 µm. ^***^ *p* < 0.001. f) Representative Western blot gel documents and summarized data showed the relative protein levels of PGC‐1α and TFAM in HK‐2 with aristolochic acid (AA) treatment. ^***^ *p* < 0.001. (n = 6 biologically independent experiments). g) Oxygen consumption rate (OCR), which was measured by the XFe96 Seahorse analyzer, in HK‐2 cells with different treatments. (n = 3 biologically independent experiments). Data are expressed as mean ± SEM (a,c–f). Two‐way ANOVA followed by Tukey's post‐test (a,c–f).

We found that conditional knockout of *Mydgf* in the tubules reduced the mitochondrial DNA (mtDNA) copy number in the kidney from mice with AAN (Figure S4a, Supporting Information), which was counteracted by *Mydgf* overexpression (**Figure** **4**a). Transmission electron microscopy (TEM) showed that the mitochondrial cristae decreased in number, and became shorter and more disordered in alignment in the tubular epithelial cells from mice with AAN, which was partially reversed by *Mydgf* overexpression (Figure 4b). Moreover, *Mydgf* deficiency promoted the aberrant expression of several key factors involved in mitochondrial homeostasis, such as peroxisome proliferator‐activated receptor gamma co‐activator 1 alpha (PGC‐1α), mitochondrial transcription factor A (TFAM), mitofusin‐2 (MFN2) and dynamin‐related protein 1 (Drp1), in the kidney from AAN mice which were reversed in AAN mice with *Mydgf* overexpression (Figure 4c; Figure S4b, Supporting Information).

In vitro, overexpression of *MYDGF* increased mitochondrial mass, inhibited mitochondrial fission, and upregulated the expression of PGC‐1α and TFAM (Figure 4d–f). By the analysis of the mitochondrial oxygen consumption ratio (OCR), we found that *MYDGF* overexpression increased oxygen consumption in AA‐treated HK‐2 cells (Figure 4g), indicating the importance of MYDGF in maintaining mitochondrial homeostasis.

### 2.4 MYDGF Maintained Mitochondrial Homeostasis by Inducing IDH2 Expression

**Figure 5:**
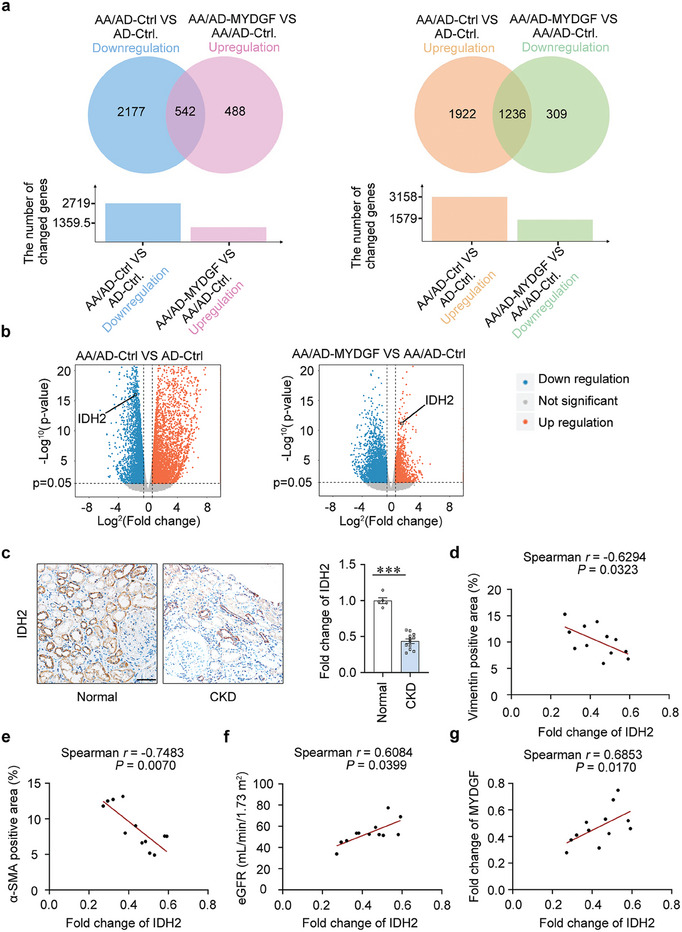
IDH2 was a target of MYDGF in tubular epithelial cells. a) Venn diagrams represented the number of differentially expressed genes in HK‐2 cells with different treatments by RNA‐seq analysis. b) Volcano plots showed the expression of *IDH2* in HK‐2 cells with different treatments. Statistically significant changes were marked to indicate whether genes are expected to increase (red) or decrease (blue). c) Photomicrographs and quantifications of IDH2 staining in kidney sections of patients with CKD. Scale bar, 50 µm. ^***^ *p* < 0.001. (n = 5 for normal participants, n = 12 for patients with CKD). d) Correlation between IDH2 expression and the degree of Vimentin staining in all participants with CKD. (n = 12). e) Correlation between IDH2 expression and the degree of α‐SMA staining in all participants with CKD. (n = 12). f) Correlation between IDH2 expression and eGFR in all participants with CKD. (n = 12). g) Correlation between IDH2 expression and MYDGF expression in all participants with CKD. (n = 12). Data are expressed as mean ± SEM (c). Two tailed Student's unpaired *t*‐test analysis (c). Spearman's correlation coefficient *r* with two‐tailed *P*‐value (d–g).

**Figure 6:**
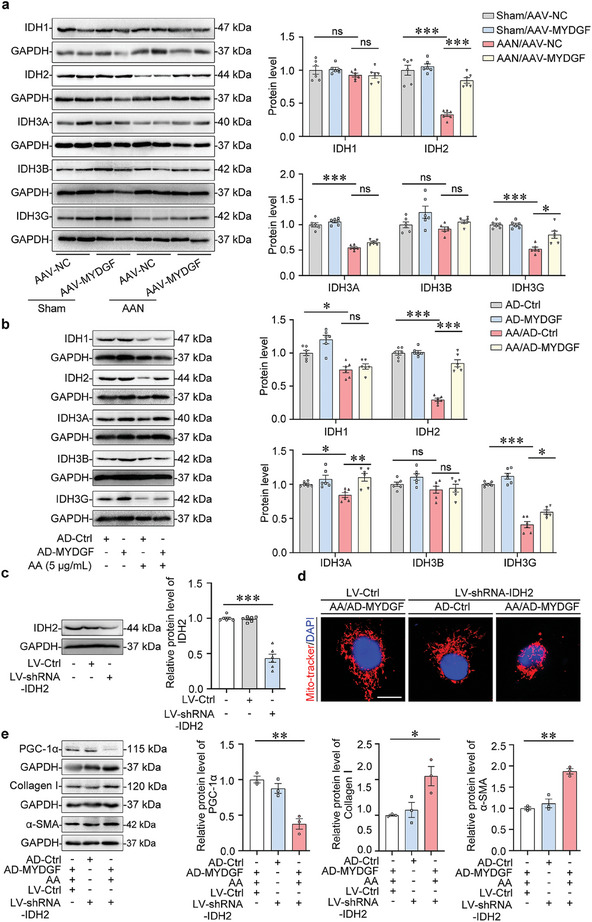
MYDGF maintained mitochondrial homeostasis by inducing IDH2 expression. a) Representative Western blot gel documents and summarized data showed the relative protein levels of IDH1, IDH2, IDH3A, IDH3B, and IDH3G in the cortex of the kidney from different groups of mice. ^*^ *p* < 0.05, ^***^ *p* < 0.001. (n = 6 mice per group). b) Representative Western blot gel documents and summarized data showed the relative protein levels of IDH1, IDH2, IDH3A, IDH3B, and IDH3G in HK‐2 with aristolochic acid (AA) treatment. ^*^ *p* < 0.05, ^**^ *p* < 0.01, ^***^ *p* < 0.001. (n = 6 biologically independent experiments). c) Gene silencing efficiency of *IDH2* by western blot analysis in shRNA‐*IDH2* transfected HK‐2 cells. ^***^ *p* < 0.001. (n = 6 biologically independent experiments). d) Representative confocal microscopy images of mitochondria stained by Mito‐tracker in different groups of HK‐2 cells. Scale bar, 10 µm. e) Representative Western blot gel documents and summarized data showed the relative protein levels of PGC‐1α, Collagen I, and α‐SMA in HK‐2 with different treatments. ^*^ *p* < 0.05, ^**^ *p* < 0.01. (n = 3 biologically independent experiments). Data are expressed as mean ± SEM (a–c,e). Two‐way ANOVA followed by Tukey's post‐test (a–c,e).

To explore the mechanism by which MYDGF maintains mitochondrial homeostasis and protects against tubular epithelial cell injury, we performed global gene expression profiling of AA‐treated HK‐2 cells with overexpressing *MYDGF*. Our results showed that *MYDGF* overexpression reversed the downregulation of 542 genes and the upregulation of 1236 genes in AA‐treated HK‐2 cells (**Figure** **5**a; Figure S5a, Supporting Information). The Kyoto Encyclopedia of Genes and Genomes (KEGG) and Gene Ontology (GO) database‐based analyses of the above genes indicated that MYDGF was mainly involved in metabolic and mitochondrial homeostasis (Figure S5b,c, Supporting Information). Among these differentially expressed genes, *IDH2*, a member of the isocitrate dehydrogenase family, was markedly reduced in AA‐treated HK‐2 cells but increased in AA‐treated HK‐2 cells with overexpressing *MYDGF* (Figure 5b). Meanwhile, KEGG and GO analyses showed that *IDH2* was involved in the glyoxylate metabolic process, glutathione metabolism, and central carbon metabolism in cancer which were related to mitochondrial homeostasis. Importantly, IDH2 was reduced in the tubules of patients with CKD (Figure 5c), and the level of IDH2 in the kidney negatively correlated with the expression of Vimentin and α‐SMA but positively correlated with MYDGF expression and eGFR (Figure 5d–g), suggesting that IDH2 might be a key target in MYDGF‐mediated mitochondrial homeostasis. Hence, we assessed the expression patterns of IDHs in the kidney from AAN mice and found that AA treatment reduced the protein levels of IDH2, IDH3A, and IDH3G, whereas the overexpression of *Mydgf* significantly upregulated the protein level of IDH2 (**Figure** **6**a). In vitro, *MYDGF* overexpression restored IDH2 expression in HK‐2 cells with AA treatment (Figure 6b). Furthermore, gene silencing of *IDH2* (Figure 6c) counteracted the protective effect of MYDGF on mitochondrial homeostasis (Figure 6d,e). In addition, gene silencing of *IDH2* contributed to the loss of the epithelial phenotype in AA‐treated HK‐2 cells overexpressing *MYDGF* as evidenced by the upregulation of Collagen I and α‐SMA (Figure 6e). These results indicate that IDH2 is a key molecule that links MYDGF to mitochondrial homeostasis in tubular epithelial cells.

### 2.5 Renoprotective Effects of MYDGF in Mice with UUO and FAN

**Figure 7:**
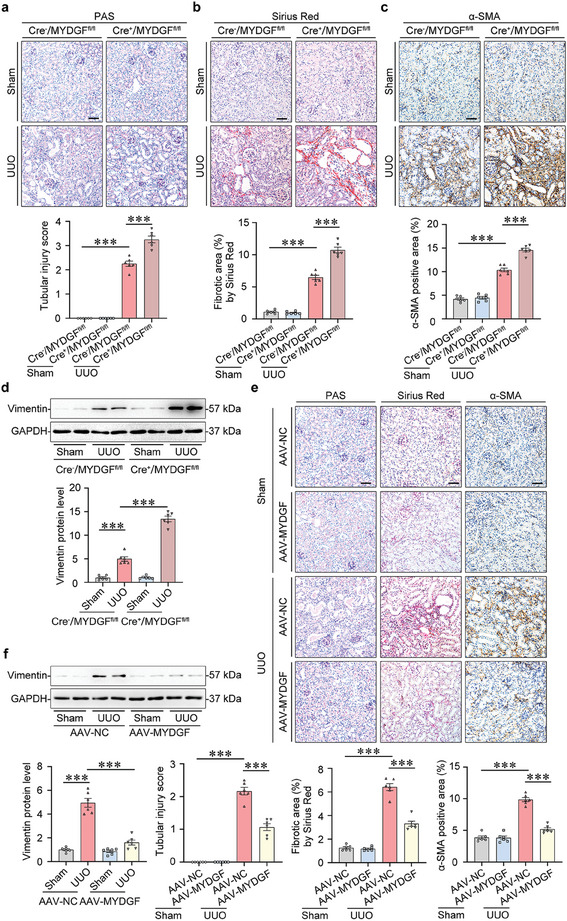
MYDGF protected against kidney injury in UUO mice. a) PAS staining was performed to assess kidney injury. Scale bar, 50 µm. ^***^ *p* < 0.001. (n = 6 mice per group). b) Sirius Red staining was performed to assess kidney fibrosis. Scale bar, 50 µm. ^***^ *p* < 0.001. (n = 6 mice per group). c) Photomicrographs and quantifications of α‐SMA staining were performed to assess kidney fibrosis. Scale bar, 50 µm. ^***^p < 0.001. (n = 6 mice per group). d) Representative Western blot gel documents and summarized data showed the relative protein levels of Vimentin in the cortex of the kidney from different groups of mice. ^***^ *p* < 0.001. (n = 6 mice per group). e) PAS staining and Sirius Red staining were performed to assess kidney injury and fibrosis. Photomicrographs and quantifications of α‐SMA staining were performed to assess kidney fibrosis. Scale bar, 50 µm. ^***^ *p* < 0.001. (n = 6 mice per group). f) Representative Western blot gel documents and summarized data showed the relative protein levels of Vimentin in the cortex of the kidney from different groups of mice. ^***^ *p* < 0.001. (n = 6 mice per group). Data are expressed as mean ± SEM (a–f). Two‐way ANOVA followed by Tukey's post‐test (a–f).

Tubule‐specific knockout of *Mydgf* aggravated tubular atrophy and kidney fibrosis in UUO mice (**Figure** **7**a,b). Moreover, *Mydgf* deficiency upregulated the expression of α‐SMA, Vimentin, Collagen I, and Collagen IV in the kidneys of UUO mice (Figure 7c,d; Figure S6a, Supporting Information). Similar results were also observed in FAN mice (Figure S7, Supporting Information). However, overexpression of *Mydgf* attenuated tubular injury and tubulointerstitial fibrosis by the PAS staining and Sirius Red staining analyses in mice with UUO (Figure 7e). At the molecular levels, *Mydgf* overexpression reduced the expressions of α‐SMA, Vimentin, and Collagen I in the kidney from UUO mice (Figure 7e,f; Figure S6b, Supporting Information), suggesting that tubular MYDGF may be a central target molecule that protects against kidney fibrosis in CKD.

## 3 Discussion

MYDGF is originally recognized as a paracrine protein mainly secreted by bone marrow‐derived monocytes and macrophages, and recombinant MYDGF has protective effects in the cardiovascular system including myocardial infarction,^[^ ^7^ ^]^ and atherosclerosis.^[^ ^10b^ ^]^ Emerging evidence has indicated that MYDGF is also expressed in parenchymal cells and plays important roles such as endothelial cells.^[^ ^13^ ^]^ In the kidney, although studies have reported the contribution of exogenous recombinant MYDGF to maintaining kidney function, several aspects need to be further considered. First, a low concentration of recombinant human MYDGF mitigated oxidative stress, inflammatory response, and apoptosis levels in I/R mice, but a high concentration of recombinant human MYDGF had no protective effects on kidney function, raising concerns about the efficiency and safety of exogenous recombinant MYDGF.^[^ ^15^ ^]^ Second, although mucin‐fused MYDGF exhibited renoprotective effects on kidney injury in CKD rats, the regulatory mechanisms and the potential targets of MYGDF under pathological conditions remain unclear.^[^ ^16^ ^]^ Particularly, we found that the level of MYDGF expression was significantly decreased in the proximal tubules of mice with CKD which was further confirmed in the tubules from CKD patients, and the level of MYDGF negatively correlated with Vimentin and α‐SMA expression but positively correlated with eGFR. Considering that tubular atrophy and tubulointerstitial fibrosis have been proven to be reliable features for the prediction of progression to end‐stage kidney disease,^[^ ^4^, ^17^ ^]^ our results suggest that MYDGF might be a potential biomarker for CKD and predict the progression of kidney damage.

It is known that the kidney has a high resting metabolic rate and is one of the most energy‐demanding organs for maintaining its functions.^[^ ^18^ ^]^ The high metabolic rate is predominantly driven by proximal tubular epithelial cells which are primarily dependent on the oxidative phosphorylation of fatty acids to meet energy demand.^[^ ^5^ ^]^ Therefore, proximal tubular epithelial cells are rich in mitochondria and are sensitive to imbalances in mitochondrial homeostasis. Importantly, accumulating evidence has also indicated that mitochondrial dysfunction is a key event in tubular injury during the progression of CKD.^[^ ^19^ ^]^ Mitochondrial dysfunction not only inhibits the supply of energy but also contributes to the generation of reactive oxygen species (ROS), inflammation, G2/M arrest, and epithelial‐mesenchymal transition, finally leading to tubular injury.^[^ ^19^, ^20^ ^]^ It is known that mitochondrial homeostasis is a dynamic process regulated by mitochondrial quality control (biogenesis), mitochondrial dynamics (fission and fusion), and energy metabolism (oxidative phosphorylation).^[^ ^21^ ^]^ Among the key regulatory factors, PGC‐1α and TFAM are important for the regulation of mitochondrial biogenesis,^[^ ^22^ ^]^ and MFN2 and Drp1 are essential for mitochondrial dynamics in tubules during the development of kidney fibrosis.^[^ ^23^ ^]^ In this study, AA treatment induced the aberrant expression of PGC‐1α, TFAM, MFN2, and Drp1, reduced mitochondrial respiration oxidative phosphorylation, and caused the loss of mitochondrial mass and the abnormality of mitochondrial morphology, which can be attenuated by *MYDGF* overexpression in tubular epithelial cells, suggesting that MYDGF is essential for the maintaining mitochondrial homeostasis in the kidney.

Mechanistically, MYDGF positively regulated the expression of IDH2, which is a member of the IDHs family.^[^ ^24^ ^]^ IDH2 is a key factor in the regulation of mitochondrial biogenesis,^[^ ^25^ ^]^ fission and fusion,^[^ ^26^ ^]^ and energy metabolism.^[^ ^27^ ^]^ Previous studies revealed that IDH2 was decreased in the proximal tubules and *Idh2* deficiency aggravated tubular injury in mice with acute kidney injury by promoting mitochondrial dysfunction,^[^ ^28^ ^]^ and exogenous gene transmission of *Idh2* protected against kidney ischemia‐reperfusion injury through restoring mitochondria activity and function,^[^ ^29^ ^]^ indicating the contribution of IDH2 to maintaining mitochondrial homeostasis in tubular epithelial cells. In this study, we demonstrated that MYDGF positively regulated the expression of IDH2 and gene silencing of *IDH2* abrogated the protective effects of MYDGF by disrupting mitochondrial homeostasis in tubular epithelial cells, suggesting that IDH2 may be a center target of MYDGF‐mediated mitochondrial function in the kidney under pathogenic conditions.

It should be noted that there are some limitations in this study. First, although we identified the role of tubular MYDGF in maintaining mitochondrial homeostasis, further studies are required to delineate the mechanisms by which various stimuli reduce MYDGF expression under pathological conditions. Second, the exact mechanism underlying MYDGF‐mediated IDH2 expression has not yet been identified. Regarding this issue, recent studies reported that *IDH2* transcription was regulated by Akt‐mediated nuclear factor erythroid 2‐related factor 2 (NRF2) signaling.^[^ ^26^, ^30^ ^]^ Considering that MYDGF can promote the activation of Akt signaling, thus MYDGF might regulate the transcription of *IDH2* by the Akt‐NRF2 axis. In addition, MYDGF can also regulate cardiomyocyte proliferation by activating forkhead box protein M1 (FoxM1), which contributes to the activation of AMP‐activated protein kinase (AMPK).^[^ ^13^, ^31^ ^]^ Notably, *IDH2* is transcriptionally upregulated by the AMPK‐forkhead box protein O1 (FoxO1) signaling pathway,^[^ ^32^ ^]^ indicating that FoxM1/AMPK/FoxO1 signaling may also be involved in the MYDGF‐mediated *IDH2* transcription. Hence, the related regulatory mechanisms require further clarification. Finally, although we demonstrated the contribution of IDH2 to regulating mitochondrial homeostasis, we cannot exclude other regulators that may be involved in this process. Therefore, further studies on other potential targets of MYDGF are also of great interest.

**Figure 8:**
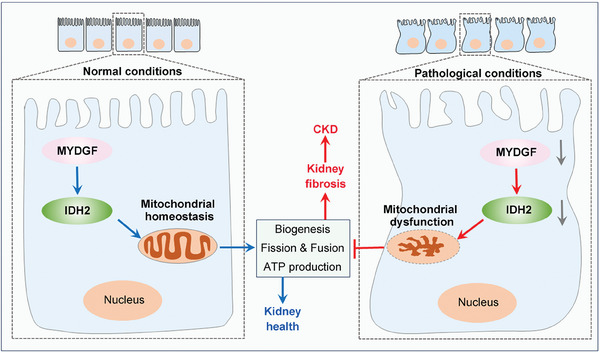
Schematic depicting tubular MYDGF slows the progression of chronic kidney disease by maintaining mitochondrial homeostasis. Under normal conditions, MYDGF maintains mitochondrial homeostasis by regulating the expression of IDH2. Under pathological conditions, MYDGF reduction leads to the downregulation of IDH2, then causes mitochondrial dysfunction by disrupting mitochondrial biogenesis, mitochondrial fission and fusion, and mitochondrial oxidative phosphorylation, finally promoting tubular injury and kidney fibrosis.

In conclusion, our study demonstrated for the first time that tubular MYDGF promotes the remodeling of mitochondrial homeostasis and alleviates kidney injury in CKD, at least in part by regulating the expression of IDH2, suggesting that targeting tubular MYDGF may be an effective therapeutic strategy for patients with CKD (**Figure** **8**).

## 4 Experimental Section

### Human Renal Biopsy Samples

Human kidney tissue samples were obtained from 12 patients who underwent renal biopsy and were confirmed to have CKD, including those with IgA nephropathy (n = 8), diabetic kidney disease (n = 3), and hypertensive nephropathy (n = 1). Normal kidney tissues were obtained from healthy kidney poles of individuals without other kidney diseases who underwent tumor nephrectomy or renal cystectomy (Table S2, Supporting Information). Kidney biopsy sections were obtained from the Department of Nephrology, Qilu Hospital, Shandong University, and the Department of Pathology, Shandong University School of Basic Medical Sciences. This study involving human samples was approved by the Research Ethics Committee of Shandong University (Document No. ECSBMSSDU2023‐1‐069) and complied with all relevant ethical regulations and guidelines of the Declaration of Helsinki. All study participants (or their parents or legal guardians) provided informed consent. None of the participants received compensation.

### Mouse Studies

All animal experiments were approved by the Institutional Animal Care and Use Committee of the School of Basic Medical Sciences, Shandong University (Document No. ECSBMSSDU2023‐2‐126) and complied with all relevant ethical regulations and the guidelines of the National Institutes of Health Guide for the Care and Use of Laboratory Animals. All mice (3–5 mice per cage) were housed under specific pathogen‐free conditions (12 h light/dark cycle, 24 °C, and 40–60% humidity) with ad libitum access to water and a standard laboratory chow diet (Beijing KEAOXIELI feed company, Beijing, China). AAN model was induced in male C57BL/6 mice (8 weeks old, 24–28 g) using a one‐time intraperitoneal injection of aristolochic acid (AA, 5 mg kg^−1^ body weight, A5512, Sigma–Aldrich) in PBS. The same amount of PBS was administered to normal control mice. For FAN, male C57BL/6 mice (8 weeks old, 24–28 g) were intraperitoneally injected with a single dose of vehicle (300 mM NaHCO_3_) or folic acid (250 mg kg^−1^ body weight, 7876, Sigma–Aldrich). UUO model (male C57BL/6 mice, 8 weeks old, 24–28 g) was established by ligating the left ureter. Sham‐operated mice subjected to the same procedure, except for ureter ligation, were used as controls.

### Generation of Tubule‐Specific Mydgf Knockout Mice

Floxed *Mydgf* mice (*B6;129S‐Mydgf^1(flox)Smoc^*, Shanghai Model Organisms Center, Inc., Shanghai, China) were backcrossed with C57BL/6 mice for more than 12 generations to produce congenic strains. C57BL/6 *Mydgf ^fl/fl^* mice were crossed with mice expressing Cre‐recombinase under the control of cadherin 16 promoter (*B6.Cg–Tg(Cdh16‐Cre)91Igr/J*, Jackson Laboratory) to generate tubule‐specific *Mydgf* knockout mice (*Cdh16‐Cre^+^*/ *Mydgf ^fl/fl^*; *Cre^+^/ Mydgf ^fl/fl^*).

### Adeno‐Associated Virus (AAV) Delivery

A tubule‐specific AAV serotype 9 was delivered harboring *Mydgf* (pAAV9‐Ksp‐cadherin‐*Mydgf*) or their negative controls produced by GeneChem Co., Ltd (Shanghai, China) through a tail vein injection into mice 4 weeks before establishing AAN and UUO models.

### Cell Culture and Treatments

Human tubule epithelial cells (HK‐2 cells) were obtained from the American Type Culture Collection and cultured in Dulbecco's Modified Eagle's Medium (DMEM) supplemented with 5% fetal bovine serum (FBS). In vitro, HK‐2 cells were serum‐starved for 12 h and then treated with 5 µg mL^−1^ aristolochic acid for 48 h. *MYDGF* was overexpressed using *MYDGF*‐adenovirus transfection (vector GV314 harboring *MYDGF*), and *IDH2* was knocked down using *IDH2*‐lentivirus transfection (vector GV493 harboring a short‐hairpin RNA sequence targeting *IDH2*) produced by GeneChem Co., Ltd (Shanghai, China).

### Oxygen Consumption Rate

The mitochondrial oxygen consumption rate (OCR) was measured using a Seahorse XFe96 flux analyzer (Seahorse Biosciences, Agilent, Santa Clara, CA, USA) with the XF Cell Mito Stress Test Kit. According to the manufacturer's protocol, 10^4^ cells per well were plated in XF‐96 extracellular flux assay plates in 80 µL of DMEM medium. After overnight incubation at 37 °C, the medium was replaced with XF DMEM (pH 7.4). The test compounds were added as follows: oligomycin (1.5 µM); FCCP (1.0 µM); rotenone/antimycin A (both 0.5 µM). The values obtained in each measurement were averaged and normalized to the number of cells per well (CellDrop BF Cell Counter, DeNovix).

### Mitochondrial DNA Copy Measurement

Total DNA was isolated from the kidneys or HK‐2 cells using a DNA extraction kit (Beyotime Biotechnology, Shanghai, China) to detect mtDNA content using real‐time PCR. 18sRNA served as a control for mtDNA. The specific primers for the mtDNA and 18sRNA in this study are listed in Table S3 (Supporting Information).

### Western Blot Analysis

Kidney tissue samples or cell pellets were lysed in RIPA buffer containing a protease inhibitor. After separation on sodium dodecyl sulfate‐polyacrylamide gel electrophoresis gels, the proteins were transferred onto polyvinylidene fluoride membranes. The antibodies used in this study are summarized in Table S4 (Supporting Information).

### Histological Analysis of Renal Tissues

Tissues were transferred to 4% paraformaldehyde, fixed at 4 °C overnight, embedded in paraffin, and cross‐sectioned (4 µm) for histological examination. Periodic acid‐achiff and Sirius Red staining were performed according to the manufacturers’ instructions (Solarbio, Beijing, China). An Olympus BX53 microscope (Olympus, Tokyo, Japan) with cellSens software was used to photograph at least six randomly chosen fields per human participant or ten randomly chosen fields per mouse within each section at 20× or 40× magnification.

### Transmission Electron Microscopy

Kidney tissues were fixed in 2.5% glutaraldehyde overnight at 4 °C, dehydrated, and embedded in Epon. The embedded tissues were cut into ultrathin sections, stained with 5% uranyl acetate and lead citrate, and analyzed using transmission electron microscopy.

### RNA‐Sequencing Analysis

The integrity of total RNA was assessed using the Agilent 2100 Bioanalyzer (Agilent Technologies Inc., USA), and samples with RNA integrity number values above 6.0 were used for sequencing. RNA (1 µg per sample) was used as the input material for library preparation. Paired‐end libraries were synthesized using the Stranded mRNA‐seq Lib Prep Kit for Illumina (ABclonal, China) following the manufacturer instructions. Purified libraries were quantified using a Qubit 3.0 Fluorometer (Life Technologies, USA) and validated using an Agilent 2100 bioanalyzer (Agilent Technologies, USA) to confirm the insert size and calculate the molar concentration. Clusters were generated using cBot with the library diluted to 10 pM and sequenced using Illumina NovaSeq 6000 (Illumina, USA). Library construction and sequencing were performed by Sinotech Genomics Co., Ltd (Shanghai, China). Paired‐end sequence files (FASTQ) were mapped to the reference genome (GRCh38.108) using Hierarchical Indexing for Spliced Alignment of Transcripts, version 2.0.5. The output sequencing alignment/map files were converted to binary alignment/map files and sorted using SAMtools (version 1.3.1). Gene abundance was expressed as fragments per kilobase of exons per million reads mapped (FPKM). Differential expression analysis of the mRNA was performed using the R package edgeR. Differentially expressed RNAs with a fold‐change ≥1.5, q <0.05, and mean FPKM >1 in one group, were considered significantly modulated and retained for further analysis. A Gene Ontology (GO) analysis and a pathway analysis was performed using the Kyoto Encyclopedia of Genes and Genomes (KEGG, http://www.genome.ad.jp/kegg) via enrich R package (version 3.4.3).

### Statistical Analysis

Data was expressed as the mean ± SEM of at least three biological replicates. Statistical analyses were performed using GraphPad Prism version 8.0 (GraphPad Software, San Diego, CA, USA). The normality assumption of the data distribution was assessed using the Kolmogorov–Smirnov test. For normally distributed data, a two‐tailed Student's *t*‐test was used to analyze the differences between the two groups. For non‐normally distributed data, the Mann–Whitney rank sum test was used to analyze the differences between the two groups. One‐way ANOVA followed by post hoc Tukey's test was used to analyze differences between multiple groups with one variable. Two‐way ANOVA followed by post hoc Tukey's test was used to compare multiple groups with more than one variable. Spearman correlation analysis was performed to assess the coefficient (*r*) and *P* value. Linear regression was performed to depict the linear relationship among variables. Spearman correlation analysis and Linear regression were performed by GraphPad Prism. All statistical details regarding *P*‐value and n can be found in main and supplementary figures legends. Significance was defined as ^*^p < 0.05, ^**^p < 0.01, ^***^p < 0.001. Different groups of mice were allocated in a randomized manner and investigators were blinded to the allocation of different groups when doing surgeries and doing outcome evaluations. The sample size in each study was based on previous experience with similar experiments in the lab. Sample sizes for each experiment were described in figure legends. Exclusion criteria were based on animal well‐being at the beginning of the study. No animals were excluded from the study.

## Conflict of Interest

The authors declare no conflict of interest.

## Author Contributions

X.L., Y.Z., Y.W., and Y.Y. contributed equally to this work. X.L., Y.Z., Y.Y., Y.W., Z.Q., and M.L. conducted in vivo and in vitro experiments, performed the data analysis and helped write the manuscript. P.Z., X.L., H.J., and Y.W. contributed to the experimental design and performed in vitro experiments. W.T., Y.S., and Y.Z. helped design experiments. X.L., Y.W., Y.Z., and Q.X. analyzed the human renal biopsy samples. M.L. and F.Y. designed the experiments, interpreted the data, and wrote the manuscript. All authors approved the final version of the manuscript for publication.

## Supporting information



Supporting Information

## Data Availability

The data that support the findings of this study are available from the corresponding author upon reasonable request.
