# Supplementary material for: Tubular MYDGF Slows Progression of Chronic Kidney Disease by Maintaining Mitochondrial Homeostasis
Source: Adv Sci (Weinh). 2024 Nov 26;12(3):2409756. doi: 10.1002/advs.202409756 (PMC11744703; doi:10.1002/advs.202409756)
Supplement: Supplementary file 1 — Supporting Information [file ADVS-12-2409756-s001.docx]

**Supporting Information**

**Tubular MYDGF Slows Progression of Chronic Kidney Disease by Maintaining** **Mitochondrial Homeostasis**

Xiaohan Liu^1#^, Yang Zhang^2#^, Youzhao Wang^1#^, Yujie Yang^1,3#^, Zhe Qiao^1^, Ping Zhan^1^, Huiying Jin^1^, Qianqian Xu^4^, Wei Tang^1^, Yu Sun^1^, Yan Zhang^1^, Fan Yi^1,5^*, Min Liu^1^*

^1^Department of Pharmacology, School of Basic Medical Sciences, Shandong University, Jinan, 250012, China.

^2^Department of Pharmacy, The Second Hospital, Cheeloo College of Medicine, Shandong University, Jinan, 250033, China.

^3^Jincheng General Hospital, Jincheng, Jincheng, 048006, China.

^4^Department of Organ Transplantation, Qilu Hospital of Shandong University, Jinan, 250012, China.

^5^National Key Laboratory for Innovation and Transformation of Luobing Theory, Key Laboratory of Cardiovascular Remodeling and Function Research, Chinese Ministry of Education and Chinese Ministry of Health, Qilu Hospital, Shandong University, Jinan, 250012, China.

^#^These authors contributed equally.

***Corresponding Author:**

Min Liu, Ph.D. Department of Pharmacology, School of Basic Medical Sciences, Shandong University, Jinan, 250012, China. Email: liuweimin@sdu.edu.cn.

Fan Yi, Ph.D. Department of Pharmacology, School of Basic Medical Sciences, Shandong University, Jinan, 250012, China. Email: fanyi@sdu.edu.cn;

**Keywords:** MYDGF, tubular epithelial cells, mitochondrial homeostasis, chronic kidney disease

**Supplementary Experimental Section**

**Mouse Studies.**

All experimental protocols for animal studies were conducted in accordance with the National Institutes of Health Guide for the Care and Use of Laboratory Animals and were approved by the Institutional Animal Care and Use Committee of School of Basic Medical Sciences, Shandong University (Document No. ECSBMSSDU2023-2-126). All mice (3-5 mice per cage) were housed under specific pathogen-free (SPF) conditions (12 h light/dark cycle, 24 °C and 40–60% humidity) with ad libitum access to water and standard laboratory chow diet (Beijing KEAOXIELI feed company, Beijing, China). Water and cages were autoclaved. Cages with standard corncob bedding were changed three times a week. For all of the *in vivo* experiments, littermate control mice were used. All of our experimental animals were kept in barrier conditions under constant veterinary supervision and did not display signs of distress or pathological changes that warranted veterinary intervention. Different groups were allocated in a randomized manner and investigators were blinded to the allocation of different groups when doing surgeries and doing outcome evaluations. The number of the mice used for the experiments is indicated for each experiment in the figure legends. An established mouse model of renal IRI was performed[1]. Briefly, male C57BL/6 mice, aged 8 weeks (24-28 g), were anesthetized with an intraperitoneal injection of pentobarbital sodium (30 mg/kg body weight) and maintained on a heat pad during surgery. A midline abdominal incision was made and bilateral renal pedicles were clipped for 32 min at 37.5 °C (bilateral IRI, BIRI). Sham operations were performed with exposure of both kidneys but without induction of ischemia. After surgery, the mice were maintained under SPF conditions (12 h light/dark cycle, 24 °C and 40-60% humidity) with ad libitum access to water and standard laboratory chow diet. The mice were euthanized and the kidney tissue samples were harvested for histopathological analysis after 4 weeks. The UUO model (male C57BL/6 mice, aged 8 weeks, 24-28 g) was generated by ligation of the left ureter[2]. Sham-operated mice were also performed with the same operation except ureter ligation and used as controls. After surgery, the mice were maintained under SPF conditions (12 h light/dark cycle, 24 °C and 40–60% humidity) with ad libitum access to water and standard laboratory chow diet. After 7 days of ureteral obstruction, the mice were euthanized and the kidney tissue samples were harvested for histopathological analysis. For the aristolochic acid nephropathy, male C57BL/6 mice, aged 8 weeks (24-28 g), were used. The animal model was induced by a one-time intraperitoneal injection of aristolochic acid (5 mg/kg body weight, A5512, Sigma-Aldrich) in PBS. The normal control mice were administered the same amount of PBS. After administration, the mice were maintained under SPF conditions (12 h light/dark cycle, 24 °C and 40–60% humidity) with ad libitum access to water and standard laboratory chow diet. The mice were euthanized and the kidney tissue samples were harvested for histopathological analysis after 28 days. For the folic acid nephropathy, male C57BL/6 mice, aged 8 weeks (24-28 g), were intraperitoneally injected with a single dose of vehicle (300 mM NaHCO_3_) or folic acid (250 mg/kg of body weight). After administration, the mice were maintained under SPF conditions (12 h light/dark cycle, 24 °C and 40–60% humidity) with ad libitum access to water and standard laboratory chow diet. The mice were euthanized and the kidney tissue samples were harvested for histopathological analysis after 28 days.

**Generation of Tubule-Specific *Mydgf* Knockout Mice.**

Floxed *Mydgf* mice (B6;129S-*Mydgf^tm1(flox)Smoc^*, Shanghai Model Organisms Center, Inc.,Shanghai, China) were backcrossed with C57BL/6 mice more than 12 generations to produce congenic strains. Then *C57BL/6 Mydgf^fl/fl^* mice were crossed with mice expressing Cre-recombinase under the cadherin 16 promoter (*B6.Cg–Tg(Cdh16-Cre)91Igr/J*, Jackson Laboratory) to generated tubule-specific *Mydgf* knockout mice (*Cdh16-Cre^+^*/*Mydgf^fl/fl^*; *Cre^+^/Mydgf^fl/fl^*). Age-matched mice without Cre (*Cdh16-Cre^-^/ Mydgf^fl/fl^*; *Cre^-^/Mydgf^fl/fl^*) were used as controls. Mouse genotyping was performed using genomic DNA isolated from mouse tails by PCR. The specific primers in this study are listed in Table S3 (Supporting Information). Flox genotyping produced 185 bp and 87 bp fragments for the mutant and wild type respectively. Wide type: only an 87 bp band; homozygous (*Mydgf^fl/fl^*): only a 185 bp band; heterozygous (*Mydgf^fl/+^*): both bands. A 420 bp band was detected in Cre positive (*Cre^+^*), but there was no band in Cre negative (*Cre^-^*).

**Western Blot Analysis.**

Kidney tissue samples or cell pellets were homogenized in RIPA buffer that contained protease inhibitor (Beyotime Biotechnology, China). The proteins were separated by SDS-PAGE and transferred onto PVDF membranes. Antibodies used in this study are summarized in Table S4 (Supporting Information). To document the loading controls, the membrane was probed with a primary antibody against housekeeping protein GAPDH. The quantitation was carried out after scanning and analyzed using Image J software (National Institutes of Health, Bethesda, USA). Band intensity normalized to an appropriate loading control (the housekeeping gene GAPDH) and relative abundance was presented.

**RNA Extraction and Real-Time qRT-PCR.**

Total RNA was isolated from the tissues or cells using TRIzol reagent (Invitrogen, Carlsbad, CA, USA). Real-time quantitative RT-PCR (qRT-PCR) was performed using the UltraSYBR Mixture (CWBIO, Beijing, China). Bio-Rad iCycler system (Bio-Rad, Hercules, CA, USA) with Bio-Rad CFX Manager 2.1 software (Bio-Rad, California, USA) was used to analyze the mRNA levels for target genes. Levels of the housekeeping gene *β-actin* were used as an internal control. The specific primers for target genes in this study are listed in Table S3 (Supporting Information).

**Histological Analysis of Renal Tissues.**

Tissues were transferred to 4% paraformaldehyde (PFA) and fixed at 4 °C overnight, then embedded in paraffin and cross-sectioned (4 μm) for histology examination. Periodic acid-achiff and Sirius Red staining were performed according to manufactures’ instructions (Solarbio, Beijing, China). At least six randomly chosen fields per human subject or ten randomly chosen fields per mice within each section were photographed with Olympus BX53 (Olympus, Tokyo, Japan) microscope at 20× or 40× magnification with cellSens software (Olympus, Tokyo, Japan). Quantification of collagen content after Sirius Red staining were performed by analyzing the % of staining area in randomly selected fields using the Image J software (National Institutes of Health, Bethesda, USA). Immunohistochemistry analysis was performed as described in our previous studies[3]. Antibodies used in this study are summarized in Table S4 (Supporting Information). Data are expressed as positive stained area vs. total analyzed area. All samples were examined in a blind manner.

**Immunofluorescence Staining**

Tissues were transferred to 4% PFA and fixed at 4 °C overnight, followed by paraffin embedded and cross-sectioned (4 μm) for immunofluorescence staining. Sections were incubated with different primary antibodies, and subsequently incubated with secondary Alexa 488 or 594 conjugated antibody (Abcam, Cambridge, Britain). DAPI (Roche, Mannheim, Germany) was used to stain nuclei. Antibodies used in this study are summarized in Table S4 (Supporting Information). At least eight randomly chosen fields per mice within each section were photographed with Olympus BX53 (Olympus, Tokyo, Japan) microscope at 20× or 40× magnification. The staining was quantified in positive area using the ImageJ software. *In vitro*, cells were washed in phosphate-buffered saline (PBS) three times, incubated with 200 nM MitoTracker Red (Beyotime Biotechnology, Shanghai, China) at 37 °C for 30 min, fixed with 4% paraformaldehyde for 30 min, and permeabilized with 0.1% Triton X-100 for 10 min. After washing, DAPI (Roche, Mannheim, Germany) was used to stain nuclei at room temperature. Quantitative data from at least 50 cells were determined per group from one experiment by Image J software.

**Blood Pressure Measurements in Conscious Mice**

Systolic and diastolic blood pressure were measured in mice by using the tail-cuff system (Softron BP-2010; Softron, Tokyo, Japan) as described in our previous studies[4]. Measurements were performed at day time (2:00 p.m. to 5:00 p.m.) with previous 5 days of training. The blood pressure measurements were taken three times consecutively for each mouse. The averaged data represented the blood pressure at that time point.

**References**

[1] W. Huang, B.-O. Wang, Y.-F. Hou, Y. Fu, S.-J. Cui, J.-H. Zhu, X.-Y. Zhan, R.-K. Li, W. Tang, J.-C. Wu, Z.-Y. Wang, M. Wang, X.-J. Wang, Y. Zhang, M. Liu, Y.-S. Xie, Y. Sun, F. Yi, *JCI Insight* **2022**, *7* (14), https://doi.org/10.1172/jci.insight.158571.

[2] Y. Zhang, Y. Yang, F. Yang, X. Liu, P. Zhan, J. Wu, X. Wang, Z. Wang, W. Tang, Y. Sun, Y. Zhang, Q. Xu, J. Shang, J. Zhen, M. Liu, F. Yi, *Nature Communications* **2023**, *14* (1), https://doi.org/10.1038/s41467-023-38771-4.

[3] Y. Fu, Y. Sun, M. Wang, Y. Hou, W. Huang, D. Zhou, Z. Wang, S. Yang, W. Tang, J. Zhen, Y. Li, X. Wang, M. Liu, Y. Zhang, B. Wang, G. Liu, X. Yu, J. Sun, C. Zhang, F. Yi, *Cell Metabolism* **2020**, *32* (6), 1052, https://doi.org/10.1016/j.cmet.2020.10.019.

[4] P. Zhan, Y. Zhang, W. Shi, X. Liu, Z. Qiao, Z. Wang, X. Wang, J. Wu, W. Tang, Y. Sun, Y. Zhang, J. Zhen, J. Shang, M. Liu, F. Yi, *Kidney International* **2022**, *102* (3), 546, https://doi.org/10.1016/j.kint.2022.04.027.

**Supplementary tables**

**Table S1. Physical and biochemical parameters of mice with *Mydgf* deficiency in tubules.**

| Variables | | *Cre^-^*/*Mydgf^fl/fl^* | *Cre^+^*/*Mydgf^fl/fl^* |
| --- | --- | --- | --- |
| Body weight (g) | | 26.28 ± 0.90 | 26.12 ± 1.01 |
| Kidney weight (g) | | 0.25 ± 0.01 | 0.26 ± 0.01 |
| Heart rate (beat/min) | | 499.50 ± 7.26 | 498.00 ± 9.12 |
| Blood pressure (mm Hg) | Systolic | 110.33 ± 2.39 | 110.17 ± 2.41 |
|  | Diastolic | 72.17 ± 2.41 | 72.50 ± 2.26 |

n = 6 mice per group. Data are expressed as mean ± SEM.

**Table S2. Clinical characteristics in the normal human control subjects or subjects with chronic kidney disease.**

| Normal group (n = 5) | |
| --- | --- |
| Age (years) | 33.40 ± 6.80 |
| Gender (male, n, %) | 4 (80 %) |
| SCr (μmol/L) | 66.40 ± 6.78 |
| BUN (mmol/L) | 4.91 ± 0.33 |
| eGFR (mL/min/1.73 m^2^) | 121.70 ± 10.84 |
| Subjects with renal fibrosis (n = 12) | |
| Age (years) | 44.25±5.22 |
| Gender (male, n, %) | 9 (75 %) |
| SCr (μmol/L) | 133.30 ± 7.66 (*p* = 0.0001)^*^ |
| BUN (mmol/L) | 8.96 ± 0.89 (*p* = 0.0009)^*^ |
| eGFR (mL/min/1.73 m^2^) | 53.80 ± 3.21 (*p* = 0.0003)^#^ |
| Pathological diagnosis | N (%) |
| IgA nephropathy | 8 (66.70 %) |
| Hypertensive nephropathy | 1 (8.30 %) |
| Diabetic kidney disease | 3 (25 %) |

SCr, serum creatinine; BUN, blood urea nitrogen; eGFR, estimated glomerular filtration rate; # Mann-Whitney rank sum test with two-tailed *p*-value; * Unpaired t-test with two-tailed *p*-value; SEM, Standard error of mean; Data are expressed as mean ± SEM.

**Table S3.** **Primer pairs of target genes used for PCR in this study.**

| Gene | Species | Primer sequences |
| --- | --- | --- |
| *Mydgf-LoxP* | Mouse | Forward: CTGAAGTGAGTCCGGGAGC  Reverse: TGTTTGGGTTGGAGTTTGC |
| *Cdh16-Cre* | Mouse | Forward:  P1: GCAGATCTGGCTCTCCAAAG  P3: CAAATGTTGCTTGTCTGGTG  Reverse:  P2: AGGCAAATTTTGGTGTACGG  P4: GTCAGTCGAGTGCACAGTTT |
| *Collagen I* | Mouse | Forward: TGACTGGAAGAGCGGAGAGT  Reverse: GTTCGGGCTGATGTACCAG |
| *Fibronectin* | Mouse | Forward: CGAGGTGACAGAGACCACAA  Reverse: CTGGAGTCAAGCCAGACACA |
| *mtDNA* | Mouse | Forward: CGATTCTTTACCTTTCACTTCATCTT  Reverse: GAGGGCGTCTTTGATTGTGT |
| *mtDNA* | Human | Forward: GGCACATGCAGCGCAAGTAGG  Reverse: GGCGGGCAGGATAGTTCAGACG |
| *β-actin* | Mouse | Forward: GGCTGTATTCCCCTCCATCG  Reverse: CCAGTTGGTAACAATGCCATGT |
| *18sRNA* | Mouse | Forward: GCGGTTCTATTTTGTTGGTTTT  Reverse: ACCTCCGACTTTCGTTCTTG |
| *18sRNA* | Human | Forward: CAGCCACCCGAGATTGAGCA  Reverse: TAGTAGCGACGGGCGGTGTG |

**Table S4. Antibodies used in this study.**

| Antibodies | Source | Provider | Catalog | Application |
| --- | --- | --- | --- | --- |
| MYDGF | Rabbit | Proteintech | 11353-1-AP | IF, IHC, WB |
| Vimentin | Rabbit | Abcam | ab92547 | WB |
| α-SMA | Rabbit | Abcam | ab124964 | IHC, WB |
| Collagen I | Rabbit | Cell Signaling Technology | 72026 | IHC |
| Collagen I | Rabbit | Abcam | ab270993 | WB |
| Collagen Ⅳ | Rabbit | Abcam | ab236640 | IHC |
| Fibronectin | Rabbit | Abcam | ab2413 | IHC |
| GAPDH | Rabbit | Abways | AB0037 | WB |
| MFN2 | Rabbit | Proteintech | 12186-1-AP | WB |
| Drp1 | Rabbit | Abcam | ab184247 | WB |
| TFAM | Rabbit | Proteintech | 22586-1-AP | WB |
| PGC-1α | Mouse | Santa | sc-517380 | WB |
| IDH1 | Rabbit | Proteintech | 12332-1-AP | WB |
| IDH2 | Rabbit | Proteintech | 15932-1-AP | WB |
| IDH3A | Rabbit | Proteintech | 15909-1-AP | WB |
| IDH3B | Rabbit | ABclonal | A13742 | WB |
| IDH3G | Rabbit | Proteintech | 25848-1-AP | WB |
| Calbindin D28k | Mouse | Santa | sc-365360 | IF |
| AQP1 | Mouse | Abcam | ab9566 | IF |

**Supplementary figures**


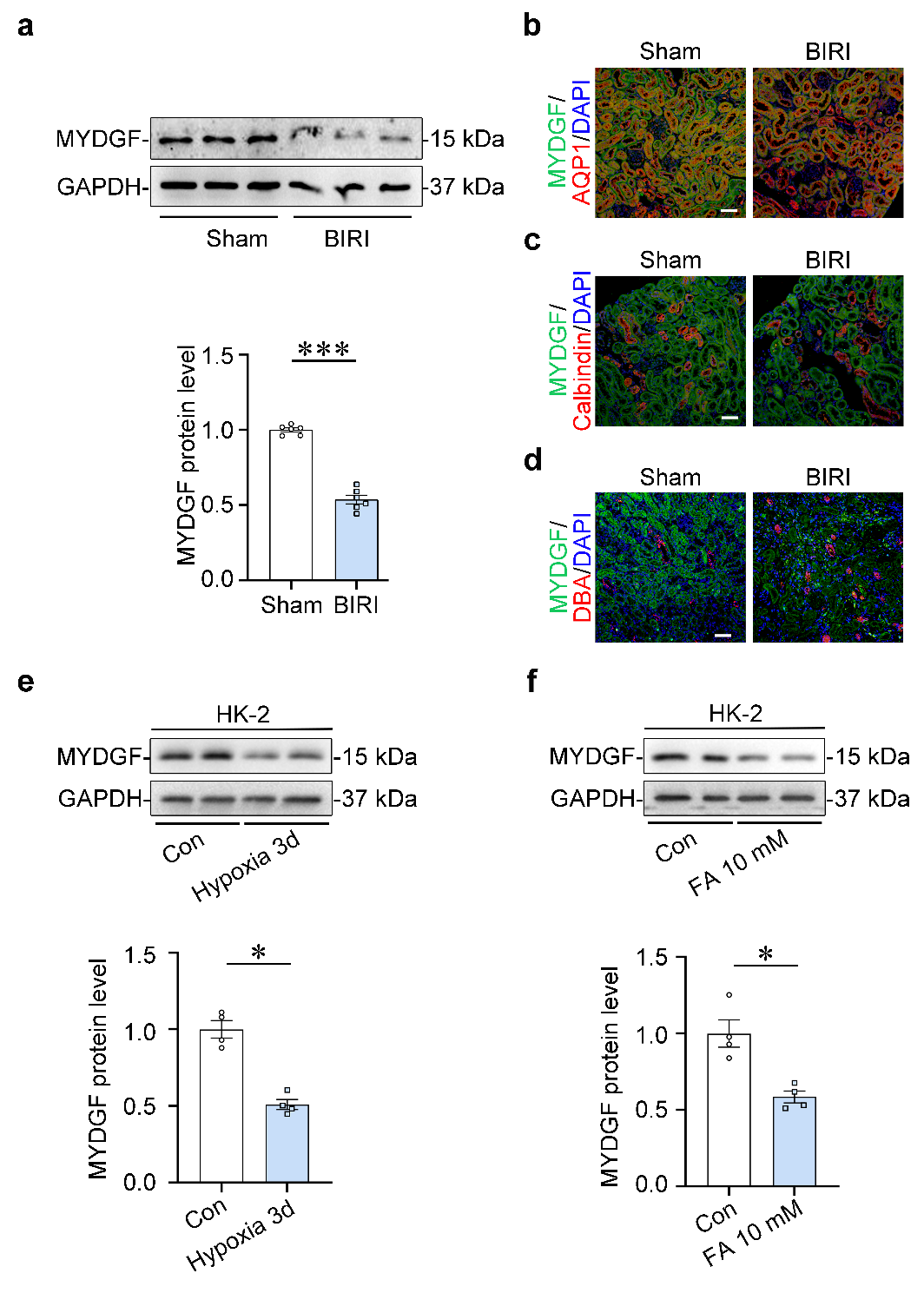


**Figure S1.** **MYDGF was significantly reduced in tubules from BIRI mice and in HK-2 cells with different treatments. a.** Protein levels of MYDGF in the cortex of kidney from BIRI mice. ****P* < 0.001. (n = 6 mice per group). **b.** MYDGF expression in tubules from BIRI mice. Aquaporin 1 (AQP1) was used as a proximal tubular marker. Scale bar, 50 μm. **c.** MYDGF expression in tubules from BIRI mice. Calbindin D28k was used as a marker for distal convoluted tubule. Scale bar, 50 μm. **d.** MYDGF expression in tubules from BIRI mice. Dolichos biflorus agglutinin (DBA) was used as a marker for collecting duct. Scale bar, 50 μm. **e.** Protein levels of MYDGF in human tubule epithelial cells (HK-2) with hypoxia treatment for 3 days. **P* < 0.05. (n = 4 biologically independent experiments). **f.** Protein levels of MYDGF in human tubule epithelial cells (HK-2) with folic acid (FA) treatment for 72 h. **P* < 0.05. (n = 4 biologically independent experiments). Data are expressed as mean ± SEM (a and e-f). Two tailed Student’s unpaired t test analysis (a). Mann-Whitney test analysis (e-f).


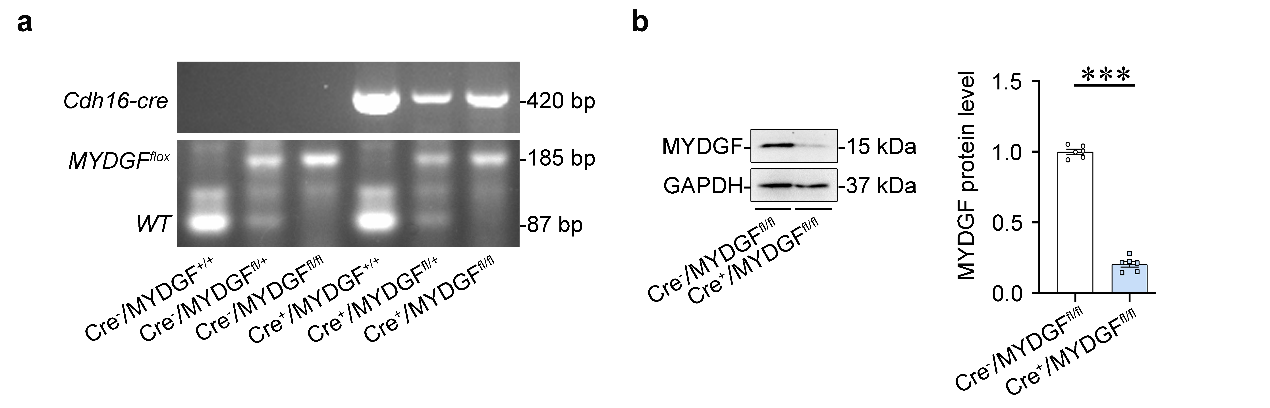


**Figure S2. Establishment of tubule-specific *Mydgf* knockout (*Cre^+^/Mydgf^fl/fl^*) mice.** **a.** Genotyping was confirmed by tail preparation and PCR at 2 weeks of age. **b.** Representative Western blot gel documents and summarized data showed the relative protein levels of MYDGF in the cortex of kidney from *Cre^+^/Mydgf^fl/fl^* mice. ****P* < 0.001. (n = 6 mice per group). Data are expressed as mean ± SEM (b). Two tailed Student’s unpaired t test analysis (b).


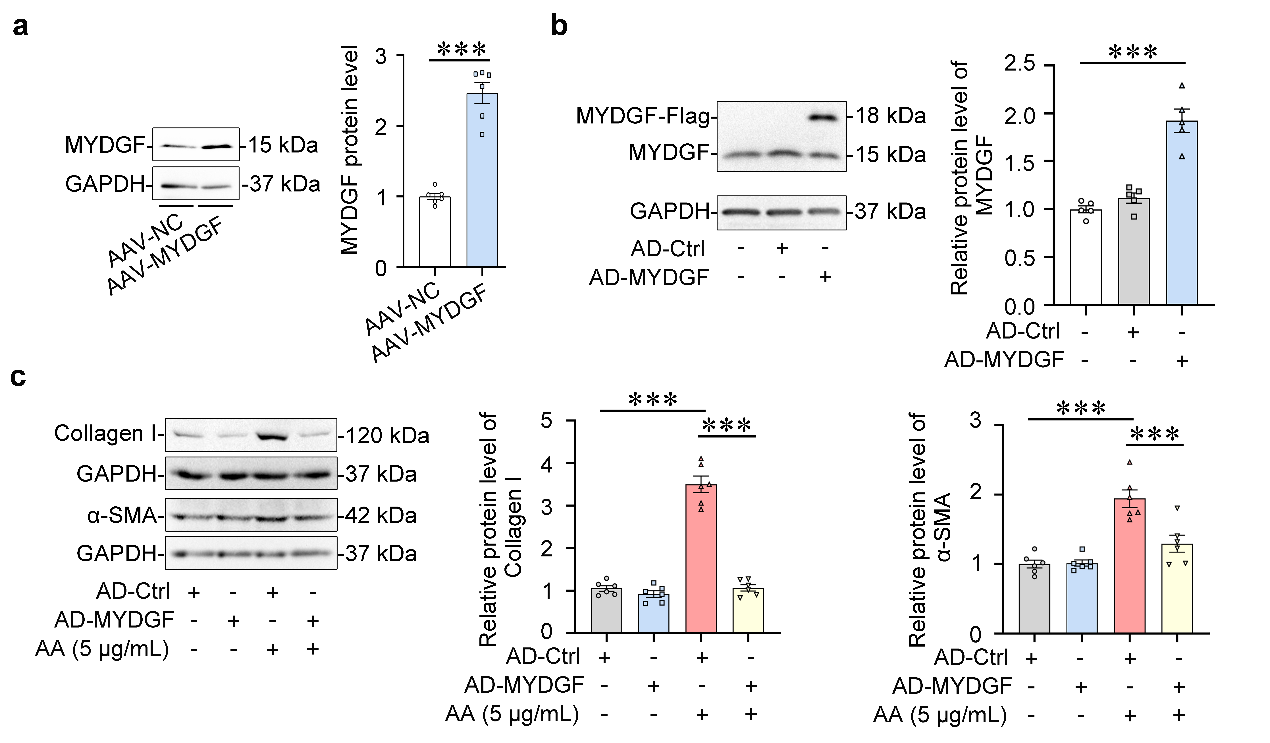


**Figure S3. Overexpression of *MYDGF* attenuated the loss of epithelial phenotype in HK-2 cells with aristolochic acid treatment. a.** Representative Western blot gel documents and summarized data showed the relative protein levels of MYDGF in kidney cortex. ****P* < 0.001. (n = 6 mice per group). **b.** Gene overexpression efficiency of *MYDGF* by western blot analysis in HK-2 cells. ****P* < 0.001. (n = 5 biologically independent experiments). **c.** Representative Western blot gel documents and summarized data showed the relative protein levels of Collagen I and α-SMA in HK-2 cells with aristolochic acid (AA) treatment. ****P* < 0.001. (n = 6 biologically independent experiments). Data are expressed as mean ± SEM (a-c). Two tailed Student’s unpaired t test analysis (a). Two-way ANOVA followed by Tukey’s post-test (b-c).


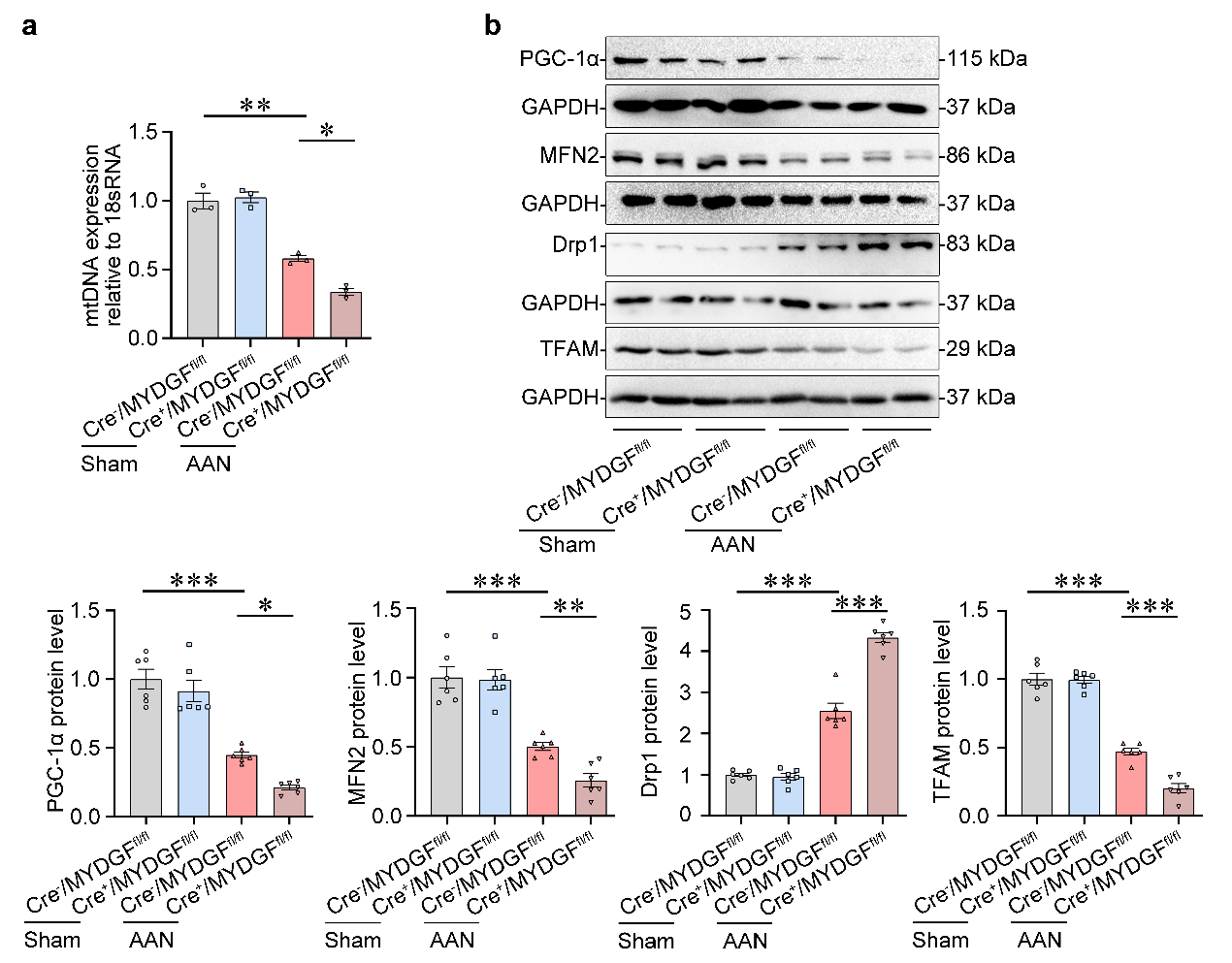


**Figure S4. Tubule-specific deletion of *Mydgf* exacerbated mitochondrial injury in mice with CKD.** **a.** Quantitative analysis of mtDNA in the cortex of kidney from different groups of mice. **P* < 0.05, ***P* < 0.01. (n = 3 mice per group). **b.** Representative Western blot gel documents and summarized data showed the relative protein levels of PGC-1α, MFN2, Drp1 and TFAM in the cortex of kidney from different groups of mice. **P* < 0.05, ***P* < 0.01, ****P* < 0.001. (n = 6 mice per group). Data are expressed as mean ± SEM (a-b). Two-way ANOVA followed by Tukey’s post-test (a-b).


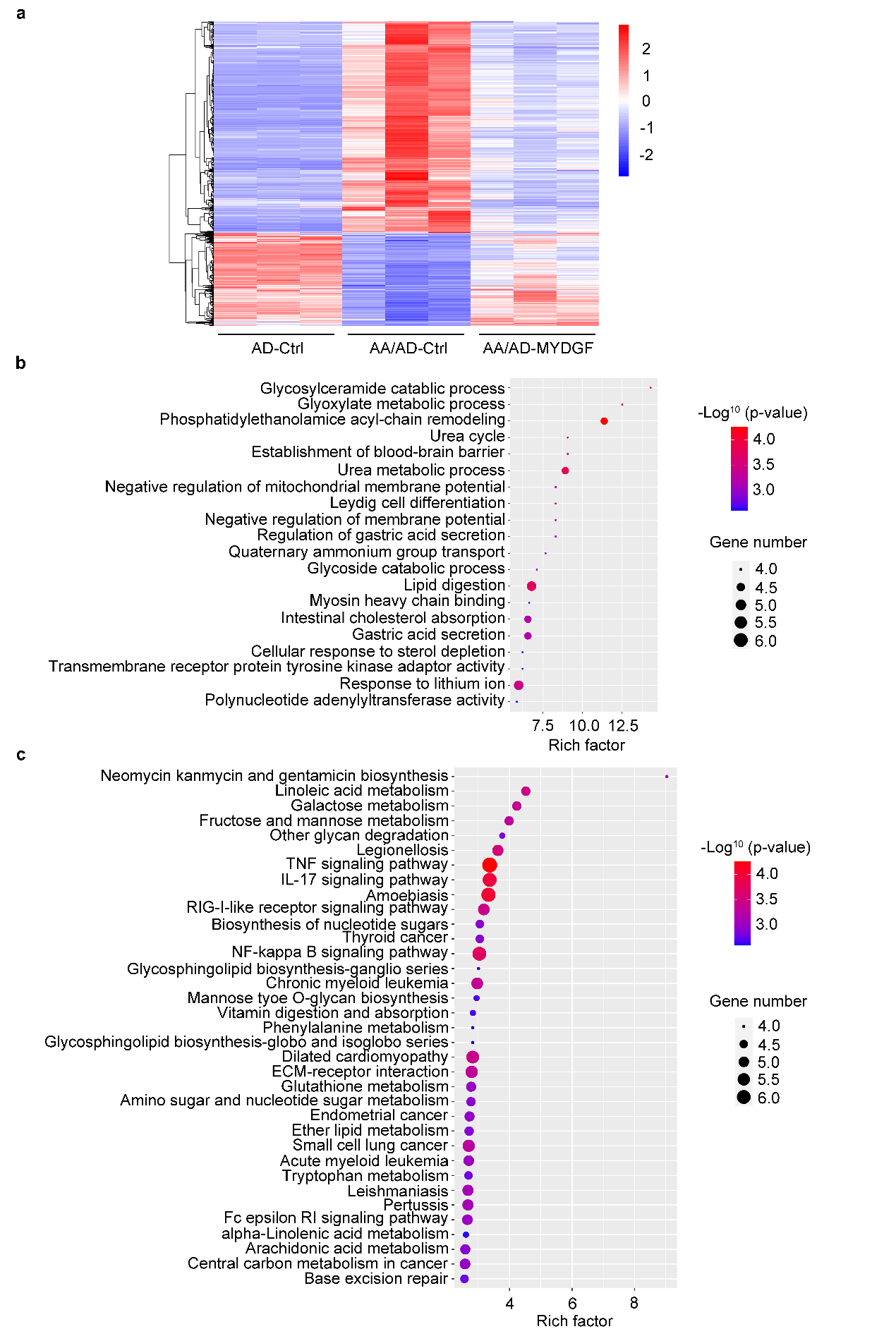


**Figure S5. Gene enrichment analysis in HK-2 with different treatments. a.** Heatmap of increased (red) or decreased (blue) genes in HK-2 cells with different treatments by RNA-seq analysis. **b.** The Gene Ontology (GO) enrichment analysis of significantly expressed genes. The bubble graphs of each presented top 20 most functionally enriched pathways. **c.** The Kyoto Encyclopedia of Genes and Genomes (KEGG) pathway enrichment analysis of significantly expressed genes. The bubble graphs of each presented top 35 most functionally enriched pathways.


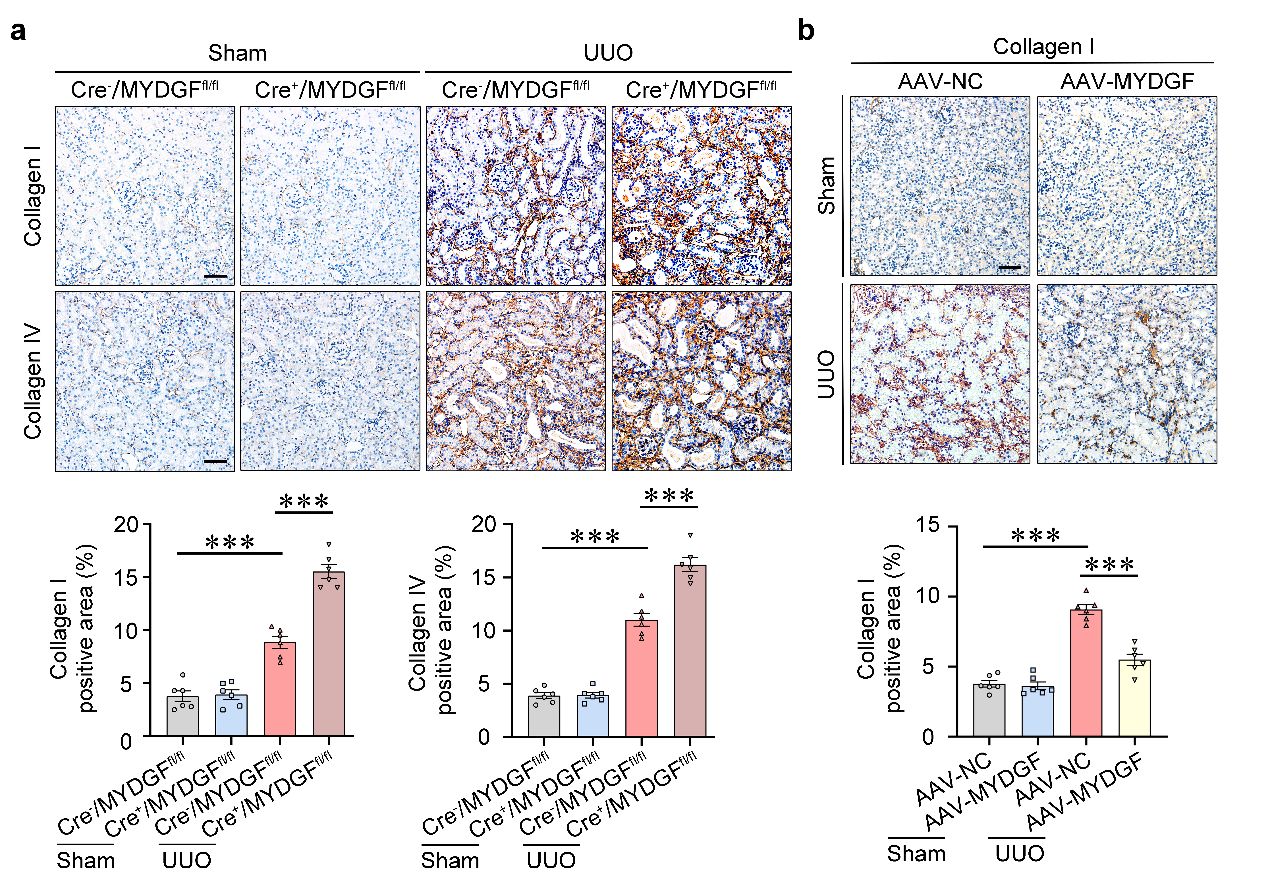


**Figure S6. MYDGF negatively regulated the expression of Collagen I and Collagen IV in the kidneys of UUO mice. a.** Photomicrographs and quantifications of Collagen I staining and Collagen IV staining were performed to assess kidney fibrosis. Scale bar, 50 μm. ****P* < 0.001. (n = 6 mice per group). **b.** Photomicrographs and quantifications of Collagen I staining were performed to assess kidney fibrosis. Scale bar, 50 μm. ****P* < 0.001. (n = 6 mice per group). Data are expressed as mean ± SEM (a-b). Two-way ANOVA followed by Tukey’s post-test (a-b).


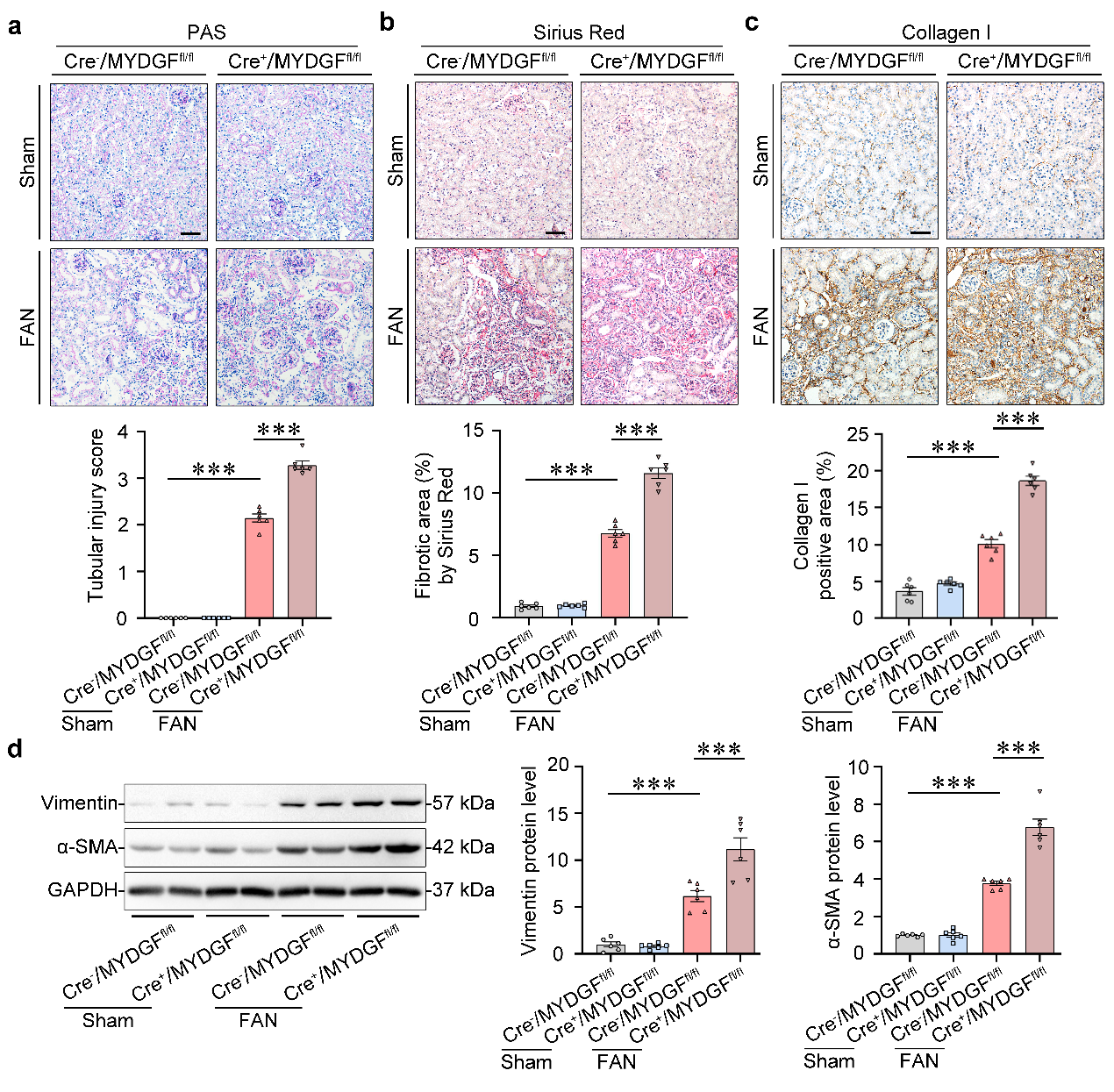


**Figure S7. *Mydgf* deficiency promoted kidney injury in mice with FAN. a.** PAS staining was performed to assess kidney injury. Scale bar, 50 μm. ****P* < 0.001. (n = 6 mice per group). **b.** Sirius Red staining was performed to assess kidney fibrosis. Scale bar, 50 μm. ****P* < 0.001. (n = 6 mice per group). **c.** Photomicrographs and quantifications of Collagen I staining were performed to assess kidney fibrosis. Scale bar, 50 μm. ****P* < 0.001. (n = 6 mice per group). **d.** Representative Western blot gel documents and summarized data showed the relative protein levels of Vimentin and α-SMA in the cortex of kidney from different groups of mice. ****P* < 0.001. (n = 6 mice per group). Data are expressed as mean ± SEM (a-d). Two-way ANOVA followed by Tukey’s post-test (a-d).
